# Targeting pain and inflammation: A comparative study of photobiomodulation with 532 and 660 nm lasers in rats

**DOI:** 10.1111/php.70013

**Published:** 2025-07-21

**Authors:** Andréa Ribeiro Mattoso‐Câmara, Juliana Zampoli Boava Papini, Marcos Aurélio Teixeira, Denise Nami Fujii, Giovana Radomille Tofoli, Aguinaldo Silva Garcez

**Affiliations:** ^1^ Faculdade São Leopoldo Mandic Campinas SP Brazil

**Keywords:** analgesic efffect, inflammatory cytokines, low power laser

## Abstract

This study evaluated the analgesic and anti‐inflammatory effects of photobiomodulation (PBM) using 532 nm (green) and 660 nm (red) low‐power lasers in an animal model of acute postoperative pain. Forty‐five Wistar rats underwent a 1 cm surgical incision on the right hind paw and were randomly assigned to three groups: red laser (RL, 660 nm, 100 mW, 5 J, 167 J/cm^2^), green laser (GL, 532 nm, 70 mW, 4.97 J, 166 J/cm^2^), and control (LO, no irradiation). PBM was applied immediately and at 1‐, 3‐, 6‐, and 24‐h postsurgery, and pain levels were assessed using von Frey's electronic analgesimeter. Inflammatory cytokines (TNF‐α, IL‐1β, CGRP, and Substance P) were measured by ELISA. Results showed that both RL and GL were significantly more effective than the control group in reducing pain and inflammation. RL provided superior analgesia, increasing pain tolerance to 690.54 ± 50.20 g at 24 h, reaching levels comparable to the non‐incised paw (*p* < 0.001). GL demonstrated greater anti‐inflammatory effects, significantly reducing TNF‐α levels at 1 h (*p* < 0.05) and 24 h and maintaining lower IL‐1β and CGRP levels. RL also modulated Substance P levels, correlating with its stronger analgesic effect. These findings suggest that RL is preferable for direct pain relief, while GL is more effective in modulating inflammatory responses. Given the statistically significant improvement in pain control and inflammatory marker modulation, PBM using these wavelengths could be a valuable adjunct therapy for postoperative pain management and enhanced healing in surgical patients. Future studies should explore synergistic PBM protocols combining both wavelengths to optimize clinical outcomes.

## Graphical abstract

This study evaluated the analgesic and inflammatory modulation effect of PBM using 660 nm (red) and 532 nm (green) lasers in a rat model of postoperative pain. Wistar rats underwent paw incision and received laser irradiation according to their groups. The red laser promoted greater analgesia, while the green laser significantly reduced TNF‐α, IL‐1β, and CGRP levels. Both wavelengths outperformed the control group, showing distinct biological effects. These findings suggest that combining different wavelengths may optimize therapeutic protocols for pain relief and inflammation modulation in surgical settings.

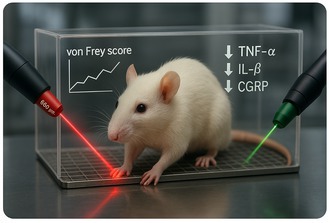

**Abbreviations**
ATP: adenosie triphosphate
AUC: area under the curve
CGRP: calcitonin gene‐related peptide
GL: green laser
IL‐1b: interleukin 1beta
LO: laser off
PBM: photobiomodulation
RL: Red laser
SP: substance P
TNF‐a: tumor necrosis factor alpha
TRPV: transient receptor potential cation channel

## INTRODUCTION

Pain is a complex, multidimensional, and subjective phenomenon that includes biological, psychosocial, and psychosomatic dimensions.^1^, ^2^ It is associated with tissue damage caused by chemical and physical agents, as well as subjective and psychological aspects. High interindividual variability is observed, requiring detailed assessment including an appropriate physical examination, history of specific pain, and assessment of functional impairment.^3^, ^4^

The causal association between trauma, tissue injury, inflammation, and acute pain, as well as the underlying mechanisms, allows for targeted treatment. Central and peripheral mechanisms are involved in pathogenesis, where the local inflammatory response allows for an increase in sensitivity and nociceptive dominance, resulting in transient sensitization of the central nervous system.^5^

Treatment protocols may include a variety of modalities, such as local or systemic analgesics and anti‐inflammatory medications, electrotherapy, invasive procedures, biofeedback, and low‐level laser photobiomodulation (PBM).^5^, ^6^, ^7^

Photobiomodulation (PBM) is a noninvasive therapeutic technique that involves the direct application of light to biological tissues to modulate cellular activity, promoting healing, reducing inflammation, and inducing analgesia. This process utilizes specific wavelengths of light, typically in the red (600–700 nm) and near‐infrared (700–1100 nm) spectra, which interact with endogenous chromophores, particularly cytochrome C oxidase in mitochondria, to stimulate ATP production, enhance oxidative metabolism, and modulate intracellular signaling pathways.^1^ The analgesic effects of PBM are also mediated by the modulation of neurotransmitters involved in pain perception, such as glutamate, which plays a key role in central sensitization and nociceptive transmission. PBM has been shown to reduce glutamate release, contributing to decreased excitotoxicity and neuroinflammation.^8^

In addition to its metabolic effects, PBM enhances nerve regeneration and repair by promoting axonal growth and synaptic plasticity, facilitating the recovery of damaged peripheral nerves.^9^ This is particularly relevant in the context of postoperative pain and tissue healing, as PBM accelerates wound closure, reduces inflammatory cytokine levels (e.g., TNF‐α, IL‐1β), and modulates neuropeptides such as Substance P and CGRP, leading to improved pain management.^10^ Given these mechanisms, PBM presents a valuable adjunct therapy for postsurgical pain management, reducing opioid dependence and enhancing patient recovery by improving nerve function and tissue regeneration.^11^, ^12^, ^13^

Other effects are related to the ability to modulate cellular activities, release growth factors by macrophages, increase lymphocyte proliferation and activation, keratinocyte proliferation, mast cell degranulation, and angiogenesis^14^, ^15^, ^16^

PBM is considered one of the most effective treatment modalities in painful situations in dentistry, such as in trigeminal neuralgia, tooth hypersensitivity, orthodontic discomfort, wound healing, and temporomandibular joint disorders.^8^, ^9^, ^17^ This therapy is also characterized by being noninvasive, causing few side effects, and being affordable. On the other hand, for it to be effective, it is necessary to accurately determine parameters that are still very inconsistent across the literature, such as effective dose for analgesic effect.^18^

Thus, the aim of this study was to analyze PBM in pain control in an animal model, comparing two different wavelengths, 660 and 532 nm, to expand the knowledge of the mechanism of action and pain control.

## MATERIALS AND METHODS

### Ethics committee

**FIGURE 1:**
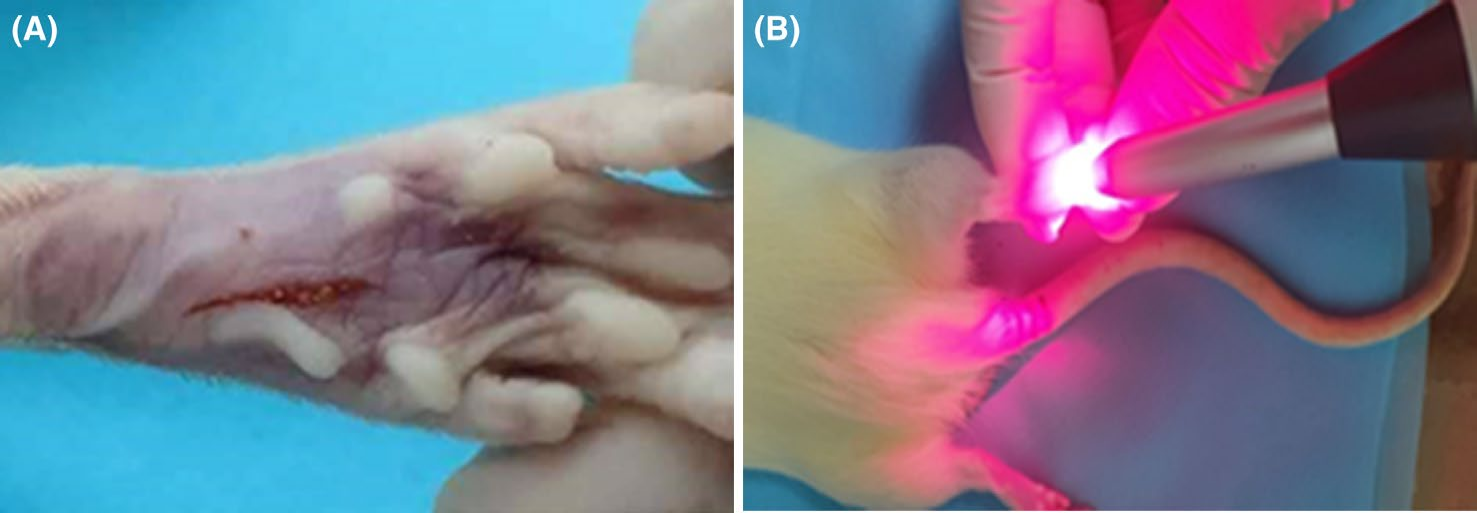
(A) Illustrative image of the surgical wound in the rat; (B) Application of the PBM.

The present study was approved by the Ethics and Animal Experimentation Committee of the São Leopoldo Mandic Institute and Research Center under protocol N^o^. 2019/036 in accordance with Law N^o^. 11794 and the standards edited by the National Council for the Control and Experimentation of Animals (CONCEA). Our research group designed it to minimize animals' stress and pain and to use the smallest possible number of animals. This study was conducted in accordance with the ARRIVE (Animal Research: Reporting of In Vivo Experiments) guidelines.^19^ Figure 1 presents a flowchart that details the group distribution and timeline of the experimental procedures. The following section provides a detailed description of the experimental protocols.

### Animal experiment

Forty‐five adult male rats (*Rattus norvegicus*, Wistar) with body weight ranging from 250 to 300 g were selected. The animals were kept in an environment with controlled temperature (22 to 25°C), fixed light‐dark cycles (12/12 h), with water and food *ad libitum*. The animals were housed in polypropylene cages (5 per cage) and acclimated to the experimental site for 7 days. The groups were chosen randomly by drawing lots. The animals were marked with surgical markers and allocated to specific cages. Thus, each group consisted of three cages, with five animals per cage.

To perform the surgical wounds, the animals were anesthetized by inhalation of isoflurane via a nasal cone as proposed by Skopelja‐Gardner et al.^20^

**FIGURE 2:**
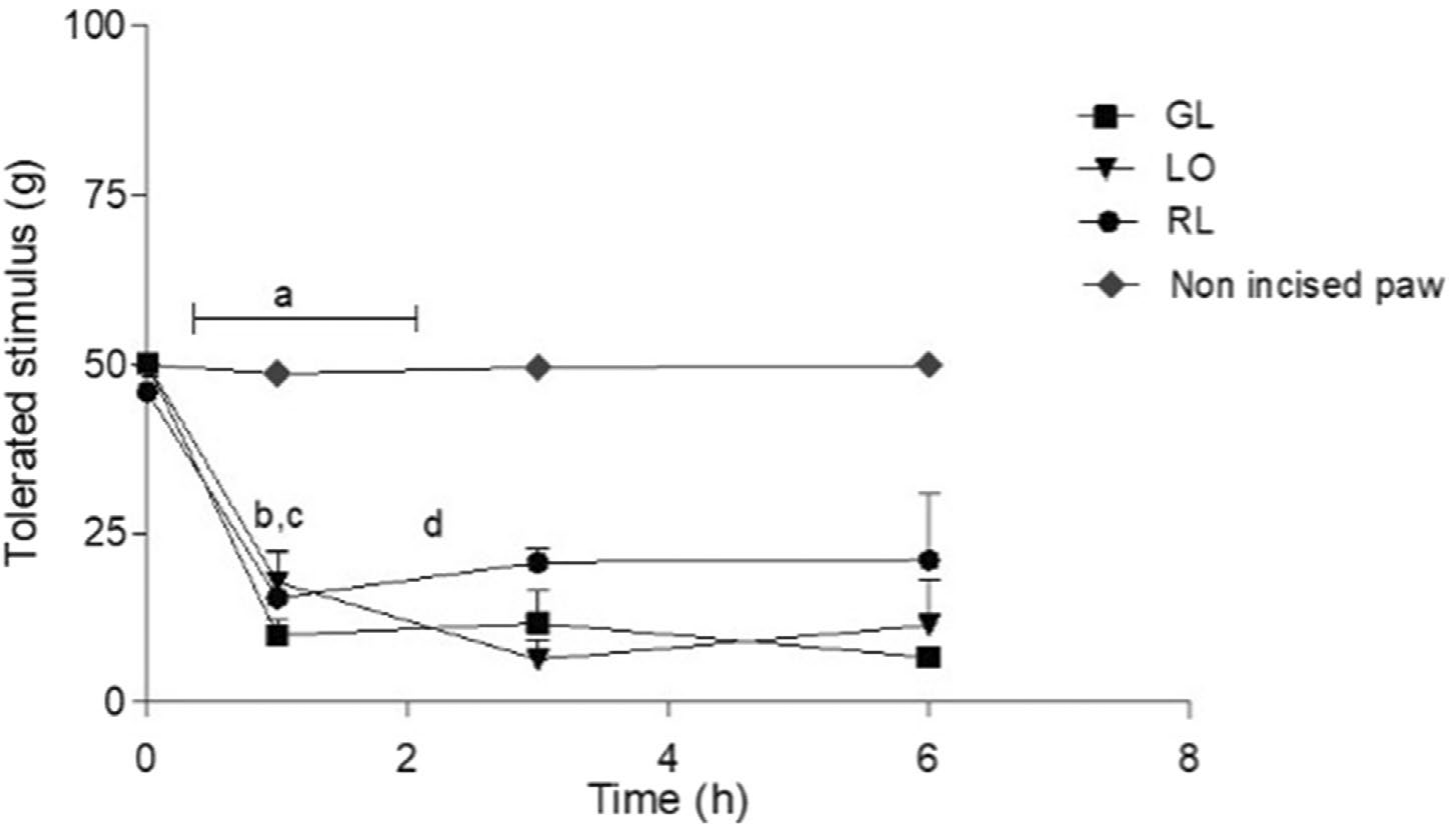
Maximum tolerated stimulus (mean ± SD) in grams after RL, GL and LO applied after at 1, 3, 6, and 24 h assessed by the von Frey test. Statistical analysis [ANOVA/Tukey–Kramer]: (A) Non‐incised paw versus GL, RL and LO (*p* < 0.001 ***) until 6 h; (B) after 3 h RL versus GL **p < 0.01 and (C) RL versus LO ***p < 0.001; (D) after 6 h RL versus **p < 0.01; (E) after 24 h RL RL versus GL, LO ***p < 0.001.

The plantar surface of the right hind paw was checked about skin pigmentation and the presence of hairs to avoid any interference during PBM irradiation. After that, they were cleaned with povidone‐iodine before and after surgery. Then, using a disposable N^o^ 11 scalpel blade, a 1‐cm longitudinal incision was made on the plantar surface of the right hind paw, starting 0.5 cm from the heel toward the base of the toe (Figure 2A) and the wound was sutured with a 5‐0 nylon suture.^17^, ^21^

In all groups, laser irradiation was performed over the surgical wound immediately after surgery (*t*0) and subsequently at 1, 3, 6, and 24 h postoperatively (Figure 2B).

After wound suture, the animals were randomly divided into three groups (*n* = 15) for treatment:

Group 1 (RL): PBM was performed with a diode laser (Laser Duo, MMOptics, Brazil) emitting at 660 nm, power output of 100 mW.
Group 2 (GL): The wounds were irradiated using a diode laser (PO®—China) emitting at 532 nm, with a power output of 70 mW.
Group 3: Laser turned off (LO) with the tip at the same position of other groups, switched off device for 50 s in the wound with 0 J/cm^2^.^18^, ^22^

To irradiate the surgical wound or to simulate the irradiation, the laser tip was positioned at one point directly over the center of the incision. The wavelength, measured power, applied energy density, and irradiation time on the surgical wound were standardized for each irradiation as presented in Table 1.

**TABLE 1:** Time/dose of lasers for surgical wound treatment.

	RL	GL	LO
Wavelength	660 nm	532 nm	0 nm
Power	100 mW	70 mW	0 mW
Power density	3.3 W/cm^2^	2.3 W/cm^2^	0 W/cm^2^
Spot size	0.03 cm^2^	0.03 cm^2^	0.03 cm^2^
Irradiation time	50 s	70 s	50 s
Energy	5 J	4.9 J	0 J
Energy density	166 J/cm^2^	163 J/cm^2^	0 J/cm^2^

### Pain assessment

The animals were housed in plastic cages suitable for the use of a von Frey analgesimeter (Dynamic Plantar Aesthesiometer®, Ugo Basile) with a wire mesh floor, in which the animals could move freely. The acclimation period was 10 min to adjust the animals' behavior before the von Frey test began.

Immediately after acclimation, an increasing force from 0 to 50 g was applied perpendicular to the plantar surface of the right and left hind paws using a single 0.5‐mm‐diameter straight metal (NiTi alloy) wire through the bottom of the cage until the response to withdrawal of the paw was recorded in grams on the device. Pain threshold was measured with three applications of force 10 min apart, and basal sensitivity was assessed considering the average of the measured forces.^23^, ^24^ Values measured after surgery that were above this threshold were considered analgesia, with 50 g considered complete analgesia.

Von Frey test was performed on the right (with surgery) and left (without surgery) hind paws, and the nociceptive behavioral response was observed as a measure of the paw withdrawal threshold. After the assessment period, the same procedure was repeated in all groups at 1, 3, 6, and 24 h after surgery. One experimenter was responsible for the paw incision and laser treatments. The examiner who performed the von Frey test was blinded to the treatments received.

The success of analgesia was defined in terms of the cutoff pressure of the von Frey analgesimeter recorded three times after the application of 50 g force at 10‐min intervals each without withdrawing the paw. Basal sensitivity was assessed by the average of the measured forces. To estimate the total analgesic effect of each treatment, we generated graphs representing tolerated stimulus versus time and calculated the area under the curve (AUC) through trapezoidal approximation, beginning at time zero and ending at 6 h. After 1‐, 6‐, and 24‐h postsurgery, five animals per group were euthanized using an overdose of isoflurane (1 mL/g). The right hind paw tissue was collected by careful dissection of the surrounding muscles and soft tissues. Each sample was individually homogenized in 500 μL of lysis buffer containing protease inhibitors (RIPA Lysis Buffer, Santa Cruz Biotechnology, Dallas, TX, USA), followed by centrifugation at 10,000 rpm for 10 min at 4°C. Total protein concentration was determined by colorimetry using the BCA protein assay kit (Thermo Scientific, Rockford, IL, USA). The resulting supernatants were stored at −20°C until analysis.

Protein levels of tumor necrosis factor‐alpha (TNF‐α), interleukin‐1β (IL‐1β), and Substance P (SP) in the right hind paw tissue were quantified using enzyme‐linked immunosorbent assay (ELISA), with kits from R&D Systems (Minneapolis, MN, USA), according to the manufacturer's instructions: TNF‐α (Cat# RTA00), IL‐1β (Cat# RLB00), and Substance P (Cat# KGE007). For calcitonin gene‐related peptide (CGRP) quantification, the kit was RayBio® CGRP‐I EIA Kit (RayBiotech, Peachtree Corners, GA, USA; Cat# EIA‐CGRP).

### Statistical analysis

The sample size was calculated using the finite population formula described by Zar (2010),^25^ considering a 95% confidence level (*z* = 1.96), 20% error margin (*d*), and 25% variance (*dp* ^2^). The formula used was: *n* = (*z* ^2^ × *dp* ^2^)/*d* ^2^ + *z* ^2^ (*dp* ^2^/*N*). Where *N* = sample size in a finite population; *z* = confidence interval (*α* = 0.05 or *z* = 1.96); *d* = error (20%); *dp* ^2^ = variance (25%). The calculation follows: *n* = (1.962 × 252)/202 + 1.962 (252/90) = 5.62. Based on this calculation, *n* = 5 animals per euthanasia time point was considered appropriate. This was further supported by similar studies^26^, ^27^ and the minimum number required for cytokine quantification via ELISA. Since the animals will be divided into three euthanasia time points, groups of 15 animals per treatment are required.

The data were analyzed to determine statistical differences by one‐way or two‐way analysis of variance (ANOVA) with a subsequent Tukey–Kramer test, according to the distribution of animals (*α* = 0.05). The software GraphPad Instat 3.0 and GraphPad Prism 6.0 were used to facilitate the analysis.

## RESULTS

**FIGURE 3:**
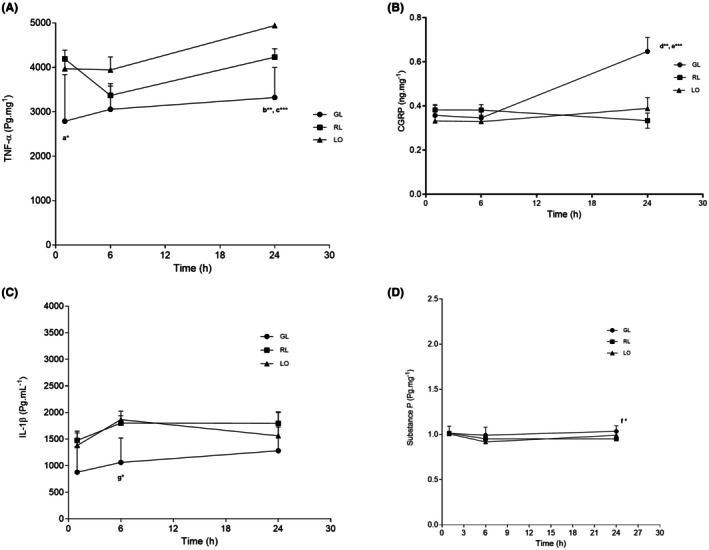
Levels in skin and plantar muscle tissue at postoperative period of 1, 6, and 24 h of TNF‐α (A); CGRP (B); Substance P (C), and IL‐1β (D). * Indicates statistically significant difference (*p* < 0.05).

The proposed animal model could promote acute inflammatory pain and hyperalgesia, as shown by the decrease in tolerated stimulus (g) after paw incision. In addition, it showed that the LO group did not exhibit an analgesic effect during all experimental periods. There was a significant difference in animals' basal levels of pain tolerance when comparing the non‐incised and the incised paw up to 6 h (*p* < 0.001). The non‐incised paw exhibited a maximum tolerated stimulus of approximately 50 g throughout the evaluation period (Figure 3). Considering the tolerated stimulus (g) after 1 h, there were no significant differences between the groups that received photobiomodulation after 1 h, all groups' paws exhibited a maximum tolerated stimulus of approximately 12 g. Three hours after surgery, the RL treatment increased the tolerated stimuli (20.7 g) when compared with GL (11.7 g) (*p* < 0.01) and LO (6.3 g) (*p* < 0.001). After 6 h RL induced higher tolerated stimulus values than GL (*p* < 0.01), mean values 21 and 6.7 g, respectively. After 24 h, there were no significant differences between non‐incised paw and RL group; in addition, RL (41 g) promoted better analgesia than GL (6 g) and LO (17g) (*p* < 0.001).

Results from the von Frey analgesimeter showed that laser application did not produce an analgesic response in the first hours after surgery in all situations. Analgesia, represented by AUC values, showed that RL was superior to GL and LO at 6 (*p* < 0.01) after laser application on the surgical wound of the animals (Table 2).

**TABLE 2:** Mean and standard deviation of the AUC of analgesia intensity by time.

Time (h)	RL	GL	LO
1	35.37 ± 3.65^a^	30.70 ± 2.15 ^a^	32.68 ± 5.47^a^
3	84.12 ± 24.20^b^	49.84 ± 6.27^c^	50.93 ± 7.27^c^
6	187.80 ± 67.89^d^	72.77 ± 11.10^e^	77.30 ± 14.39^e^

*Note*: Area under the curve in a.u. Different letters indicate statistical difference (*p* < 0.05).

The analysis of TNF‐α data showed that GL decreased TNF‐α levels at 1 h after surgery compared with RL and LO (*p* < 0.05). Six hours after incision, there was no difference between the groups. After 24 h, GL exhibited the lowest TNF‐α levels when compared with RL (*p* < 0.01) and the LO group (*p* < 0.001), as shown in Figure 3A. Considering the levels of CGRP at 1 and 6 h after incision (Figure 3B), there was no difference between groups. At 24 h, CGRP levels were higher in GL than in RL (*p* < 0.01) and Laser off (*p* < 0.001).

Figure 3C shows that IL‐1 β levels at 6 h were lower for GL compared with RL and LO (*p* < 0.05). No statistical difference was observed in other periods of the experiment, even GL levels showing lower values than RL and LO.

Substance P levels at 1 and 6 h after incision showed no difference between groups; however, at 24 h, there was a statistical difference between RL and GL, as shown in Figure 3D.

## DISCUSSION

This study assessed the levels of pain in the first postoperative periods using von Frey's electronic analgesimeter and modulations on the inflammatory process by IL‐1β and TNF‐α, which are the first proinflammatory cytokines produced where IL‐1 β induces the release of neuropeptide SP and CGRP peptide and TNF‐α stimulates the production of SP.^18^, ^19^

According to Zeng et al.,^28^ lasers emitting at red and infrared wavelengths have been widely used in medicine and dentistry for photobiomodulation therapies. However, some studies show that other wavelengths, including green light, can provide benefits such as promoting analgesia and altering the levels of TNF‐α and IL‐1β.^29^, ^30^, ^31^ Based on that principle, this study evaluates different wavelengths on the analgesic effect of PBM.

von Frey test showed significant differences between LO and the other groups—RL and GL after 6 and 24 h after PBM when irradiation was performed directly over the wound. Furthermore, RL was superior to the GL in controlling postoperative pain at 3, 6, and 24 h and showed significant levels of analgesia compared with the LO group. Similar results were found by Hu et al.^27^ using a 670 nm LED in rat spinal cord injury, which found a 40% reduction in pain hypersensitivity; also Pigatto et al.^32^ in an animal model of chronic pain found a significant reduction in pain after 24 h.

Although the experimental parameters (light source, power, energy, energy density, irradiated area, irradiation time, and wavelength) were adjusted to comparable levels, the differences observed in photobiomodulation effects can largely be attributed to the distinct ability of each wavelength to penetrate tissues and interact with chromophores.^31^, ^33^ The RL demonstrated superior analgesic effects in the von Frey test, particularly significant at 3, 6, and 24 h postsurgery, when it showed a significantly higher pain tolerance compared with both GL and LO. Notably, RL achieved pain control comparable to the non‐incised paw at 24 h, highlighting its role in resolving inflammatory pain, as also reported by Pigatto et al.^32^ On the other hand, GL, while less effective in immediate analgesia, exhibited a pronounced modulation of inflammatory markers, particularly TNF‐α, IL‐1β, and CGRP, which may explain its better performance in reducing inflammation. These findings, also reported by other authors^29^, ^34^, ^35^ suggest that while both wavelengths have distinct biological effects, RL appears more effective for direct pain relief, whereas GL may play a critical role in modulating the inflammatory response, offering a complementary therapeutic approach in managing postoperative pain.

Red and green wavelengths may present different light absorption by skin endogenous chromophores.^16^, ^27^, ^29^, ^36^, ^37^ The inflammatory cytokine analysis revealed distinct biological responses to each wavelength. GL exhibited a superior anti‐inflammatory effect, significantly reducing TNF‐α levels at 1 h (*p* < 0.05) and 24 h postirradiation, while RL also reduced TNF‐α levels but to a lesser extent (*p* < 0.05). The lower IL‐1β levels in GL‐treated groups further suggest an anti‐inflammatory role of GL therapy, as also reported by Ventura et al.^38^ However, RL was superior in modulating Substance P, which may correlate with its stronger analgesic effect.

These findings support the hypothesis that different wavelengths elicit distinct cellular responses: while RL appears to be more effective for analgesia and pain resolution, GL is more efficient in modulating the inflammatory cascade, particularly at early time points. The greater tissue penetration of RL, likely due to higher absorption by cytochrome C oxidase, may explain its superior efficacy in pain reduction, while the GL effect could be linked to modulation of calcium‐dependent ion channels (e.g., TRPV) and neurogenic inflammation. These findings reinforce the importance of considering wavelength‐specific mechanisms when selecting PBM protocols for pain management and inflammatory control.

Although IL1‐β levels were elevated in RL compared with LO at 24 h, suggesting inflammatory hyperalgesia and peripheral sensitization, RL showed analgesic effects during the von Frey test at 3, 6, and 24 h. However, Pereira et al.^21^ observed a decrease in the levels of the proinflammatory cytokine IL‐1β within 24 h, possibly due to the 830 nm wavelength used by the authors and furthermore, Shamloo et al.^39^ also found a down regulation of IL1‐β after irradiation with 640 and 880 nm.

Conversely, GL exhibited greater anti‐inflammatory effects, significantly reducing TNF‐α levels at 1 h (*p* < 0.05) and maintaining the lowest levels of TNF‐α and IL‐1β at 24 h. This aligns with previous findings by Salman et al.^34^ and Ventura et al.^38^ who suggest that green light modulates inflammation, particularly lowering proinflammatory cytokines. This effect may explain the early suppression of inflammatory cytokines observed in the GL‐treated group. Furthermore, the study by Janzadeh et al.^10^ supports the notion that PBM at 635–660 nm reduces oxidative stress and inflammation, while alternative wavelengths, including green light, may exert their effects through different molecular pathways, similar results observed by Serrage et al.^31^ and Hamblin et al.^33^

## CONCLUSION

Our findings demonstrate that both red (660 nm) and green (532 nm) laser wavelengths were more effective than the control group (LO) in promoting analgesia and modulating inflammation, reinforcing the therapeutic potential of photobiomodulation (PBM) in acute postoperative pain management. However, each wavelength exhibited distinct biological effects: RL (660 nm) provided superior analgesia, significantly increasing pain tolerance in the von Frey test at 3, 6, and 24 h. In contrast, GL (532 nm) demonstrated stronger anti‐inflammatory effects, significantly reducing TNF‐α at 1 and 24 h and maintaining lower IL‐1β levels. Both wavelengths outperformed the control group in evaluated parameters, demonstrating that PBM at either 532 or 660 nm is an effective strategy for postoperative pain management. Future research should explore synergistic PBM protocols, combining both wavelengths to optimize therapeutic outcomes.

## AUTHOR CONTRIBUTIONS

**Andréa Ribeiro Mattoso‐Câmara** has been involved in the acquisition of data and drafting the manuscript. **Juliana Zampoli Boava Papini:** acquisition of data. **Marcos Aurélio Teixeira:** acquisition of data. **Denise Nami Fujii:** revising it critically for important intellectual content. **Giovana Radomille Tofoli:** analysis and interpretation of data and methodology analysis. **Aguinaldo Silva Garcez:** analysis and interpretation of data and methodology analysis. All authors read and approved the final version of the manuscript to be published and agree to be accountable for all aspects of the work.

## CONFLICT OF INTEREST STATEMENT

The authors have stated explicitly that there are no conflicts of interest in connection with this article.

## ETHICS STATEMENT

Certificate from the Ethics Committee on the Use of Animals n°: 2019/036.

## Data Availability

The data that support the findings of this study are available on request from the corresponding author. The data are not publicly available due to privacy or ethical restrictions.
